# Immune response, proviral load and serologic markers of neurologic disease expression in HTLV-1 infection

**DOI:** 10.1186/1742-4690-11-S1-P67

**Published:** 2014-01-07

**Authors:** Silvane Santos, Cristina Toledo, Anselmo Souza, Davi Tanajura, Marshall Glesby, Edgar M Carvalho

**Affiliations:** 1 Serviço de Imunologia, Complexo Hospitalar Universitário Professor Edgard Santos, Universidade Federal da Bahia, Salvador, Bahia, Brazil; 2 Instituto Nacional de Ciência e Tecnologia de Doenças Tropicais (CNPq/MCT), Salvador, Bahia, Brazil; 3 Division of International Medicine and Infectious Diseases, Department of Medicine, Weill Medical College of Cornell University, New York, New York, USA; 4 Fogarty International Clinical Research Scholars and Fellows Program at Vanderbilt University, USA; 5 Escola Baiana de Medicina e Saúde Pública, Salvador, Bahia, Brazil

## 

High frequency of neurologic diseases including urinary symptoms of overactive bladder (OAB) have been documented in HTLV-1 infected subjects. Moreover OAB may be the initial manifestation of HTLV-1 associated myelopathy (HAM). The aim of this study was to evaluate the immune response, proviral load and plasma levels of soluble Interleukin-2 receptor (sIL-2R) and beta 2 microglobulin (β2M) in HTLV-1 carriers, patients with HTLV-1 OAB and in patients with HAM to determine if these parameters are markers of neurologic disease. Cells and serum from carriers, HTLV-1 OAB and HAM/TSP patients were used. HAM/TSP had higher levels of β2M and sIL-2R than HTLV-1 OAB and carriers. Cells from HTLV-1 OAB produced spontaneously more proinflammatory cytokines than carriers. TNF-α and IL-17 expression as well as proviral load were similar in HAM/TSP and HTLV-1 OAB and were higher than in carriers. In contrast to HAM/TSP, levels of Th1 chemokines were similar in HTLV-1 OAB and carriers. Different from HAM/TSP addition of regulatory cytokines in cell cultures from HTLV-1 OAB decreased spontaneous IFN-g. This study showed that while sIL-2R determination helps in identifying HAM/TSP, both sIL-2R and β2M did not help to monitor neurologic disease progression. Additionally we showed that HTLV-1 OAB and HAM/TSP have in common many immunological features as well as proviral load. However, as HTLV-1 OAB were still able to down regulate their inflammatory response and express levels of chemokines similar to carriers this may explain why they have not yet developed the spinal cord damage observed in HAM/TSP.

## Financial support

NIH (AI079238, K24078884), Brazilian National Research Council (CNPq).

